# FOXO1 inhibits FSL-1 regulation of integrin β6 by blocking STAT3 binding to the integrin β6 gene promoter

**DOI:** 10.3389/fcimb.2022.998693

**Published:** 2022-10-10

**Authors:** Mingyan Xu, Jie Huang, Feixiang Zhu, Kailun Shen, Fan Liu, Xiaoling Deng

**Affiliations:** ^1^ Xiamen Key Laboratory of Stomatological Disease Diagnosis and Treatment, Department of Implantology, Stomatological Hospital of Xiamen Medical College, Xiamen, China; ^2^ Engineering Research Center of Fujian University for Stomatological Biomaterials, Department of Stomatology, Xiamen Medical College, Xiamen, China; ^3^ School of Stomatology, Fujian Medical University, Fuzhou, China; ^4^ Department of Basic Medical Science, School of Medicine, Xiamen University, Xiamen, China

**Keywords:** FOXO1, FSL-1, ITGB6, STAT3, Periodontitis

## Abstract

Integrin β6 (ITGB6), an epithelial-specific receptor, is downregulated in the gingival epithelium of periodontitis and is associated with inflammation response and periodontitis development. However, the transcriptional regulatory mechanism of ITGB6 downregulation in the human gingival epithelium remains unclear. Fibroblast-stimulating lipopeptide-1 (FSL-1), an oral biofilm component, promotes an epithelial cell-driven proinflammatory response in periodontitis partially by suppressing ITGB6 expression. The aim of the current study was to investigate the transcriptional regulatory mechanism of ITGB6 inhibition by FSL-1 in human epithelial cells (HaCaT and primary human gingival epithelial cells), and to delineate the transcriptional mechanism of ITGB6 suppression in periodontitis. We found that FSL-1 inhibited ITGB6 transcription through increasing forkhead box protein O1 (FOXO1) expression and inhibiting signal transducer and activator of transcription 3 (STAT3) activation. Furthermore, FOXO1 bound to STAT3 directly, leading to decreased STAT3 phosphorylation induced by FSL-1. Consequently, the binding of phosphorylated STAT3 to the ITGB6 promoter was decreased, and ITGB6 transcription was therefore downregulated following FSL-1 stimulation. The reciprocal action of STAT3 and FOXO1 on ITGB6 downregulation was also confirmed by the immunostaining of the inflammatory epithelium associated with periodontitis. Our findings suggest that the interaction of FOXO1–STAT3 may be a useful signal target for the treatment of periodontitis.

## Introduction

Periodontitis is a chronic non-communicable disease affecting approximately 47% of US adults (Teles et al., 2022) and increasing the risk of developing chronic diseases (Zemedikun et al., 2021). Deep periodontal pockets between the gingiva and teeth, inflammation, and bone loss are characteristic of periodontitis. Periodontal microbial insults and associated proinflammatory cascades have been proposed to contribute to the pathogenesis of systemic diseases, such as Alzheimer’s disease, diabetes (Kocher et al., 2018), cardiovascular disease (Sanz et al., 2020), respiratory disease (Gomes-Filho et al., 2020), chronic renal disease (Hickey et al., 2020), and oral cancer (Teles et al., 2022).

Integrin β6 (ITGB6) is an epithelial-specific receptor that is absent from normal healthy epidermis and oral mucosa (Breuss et al., 1993), but is constitutively expressed in the healthy gingival epithelium (Meecham and Marshall, 2020). However previous research has shown that gingival epithelial cells (GECs) with reduced ITGB6 expression exhibit an enhanced inflammatory response (Bi et al., 2017). Moreover, the absence of ITGB6 was linked to the initiation and progression of periodontitis in a mouse model (Ghannad et al., 2008). In addition, patients with ITGB6 mutations can develop severe periodontal disease (Ansar et al., 2016). Therefore, repression of ITGB6 may play a key role in acute inflammation and periodontitis development.

Dysbiotic bacterial dental plaque biofilms are considered the main cause of periodontitis and ITGB6 suppression (Bi et al., 2019). ITGB6 expression is transcriptionally regulated. We previously identified several transcriptional binding sites for transcription factors, such as signal transducer and activator of transcription (STAT) and forkhead box-O (FOXO) in the core promoter of ITGB6 (Xu et al., 2015). However, the regulatory mechanisms underlying the repression of ITGB6 expression by oral biofilm components in periodontitis remain unexplored.

The transcription factor family FOXO includes four members: FOXO1, 3, 4, and 6 (van der Vos and Coffer, 2008; Maiese et al., 2009). FOXO proteins are increasingly considered to be unique cellular targets that regulate several cellular processes, including cell differentiation, immune status, apoptosis, and inflammation (Maiese et al., 2009). FOXO1 has been linked to the development of periodontitis. FOXO1 mediates the inflammatory effect of prostaglandins (Pg) in GECs, as FOXO1 silencing attenuates Pg-induced production and secretion of IL-1β (Wang et al., 2015). The FOXO1 signaling axis can regulate periodontal bacteria–epithelial interactions, immune-inflammatory responses, bone remodeling, and wound healing (Ren et al., 2021).

Fibroblast-stimulating lipopeptide-1 (FSL-1) is a diacylated lipopeptide mimicking the 44 kDa lipoprotein of *Mycoplasma salivarium* and is suspected to play an etiological role in periodontal diseases (Shibata et al., 2000). Inflammatory cytokines such as monocyte chemotactic protein (MCP)-1, MMP-9, IL-6, tumor necrosis factor (TNF)-α, and IL-8 can be induced by FSL-1 in monocytes/macrophages (Ahmad et al., 2014). Studies have confirmed that biofilm components and FSL-1 can downregulate ITGB6 expression in the pocket epithelium, thus, promoting an epithelial cell-driven proinflammatory response in periodontal disease (Li et al., 2013; Bi et al., 2017). However, the transcriptional regulatory mechanism of ITGB6 inhibition by FSL-1 in the human gingival epithelium remains unclear.

In the present study, we investigated the transcriptional regulatory mechanism of ITGB6 inhibition by FSL-1 in human epithelial cells (HaCaT and primary human GECs) and delineated the transcriptional mechanism of ITGB6 suppression in periodontitis. We identified that the FOXO1–STAT3 interaction may potentially contribute to FSL-1-suppressed ITGB6 expression in the gingival epithelium of periodontitis.

## Materials and methods

### Cell culture and FSL-1 treatment

The human keratinocyte cell line HaCaT was purchased from the Kunming Cell Bank of the Chinese Academy of Sciences. HaCaT cells were maintained in Dulbecco’s modified Eagle’s medium (Gibco) supplemented with 10% fetal bovine serum (Gibco) and 1% penicillin–streptomycin at 37°C in a cell incubator containing 5% CO_2_. Primary human GECs were grown from explants of gingival biopsies obtained from healthy volunteers with no gingival inflammation, as described previously (Kedjarune et al., 2001). Primary GECs were maintained in keratinocytes (K)-SFM medium (Gibco) supplemented with human keratinocytes growth supplement, 1% antibiotics, and 10 μM Y-27632 (Sigma-Aldrich, Shanghai, China). When the GECs reached 80% confluence, subculturing was performed *via* trypsin digestion. A third generation of cells was used in the experiment. Primary cells within six generations were used in this study. HaCaT and GECs were stimulated with FSL-1 (Abcam, Shanghai, China) for indicated times at 37°C. Cells were harvested for detection of ITGB6 mRNA and protein levels.

### Reverse transcription quantitative polymerase chain reaction (RT-qPCR)

Total RNA was extracted from HaCaT cells and GECs using a generic RNA extraction kit (Dongsheng Biotechnology, Hangzhou, China). Reverse transcription into cDNA was performed according to the instructions of the TaKaRa PrimeScript™ RT Reagent KIT (Takara, Dalian, China). The RT- qPCR assay was performed in ABI 7500 (Applied Biosystems, USA) using SYBR Green. The obtained data were analyzed using a comparison threshold cycle (2^−ΔΔCt^) to indicate relative mRNA expression, and GAPDH was used as an internal control. The following primers were used for PCR: ITGB6 forward, 5′- GCAAGCTGCTGTGTGTAAGGAA-3′; ITGB6 reverse, 5′-CTTGGGTTACAGCGAAGATCAA-3′; FOXO3 forward, 5′- GCGTGCCCTACTTCAAGGATAAG-3′; FOXO3 reverse, 5′-GACCCGCATGAATCGACTAATG-3′; STAT3 forward, 5′- GAGCTGCACCTGATCACCTT-3′; STAT3 reverse, 5′-CTACCTGGGTCAGCTTCAGG-3′; FOXO1 forward, 5′- GTTGCTGACTTCTGACTCT-3′; FOXO1 reverse, 5′-GCTGCCATAGGTTGACAT-3′; GAPDH forward, 5′- CATCACCATCTTCCAGGAG-3′; and GAPDH reverse, 5′-AGGCTGTTGTCATACTTCTC-3′.

### Western blotting

The cells were collected in a 1.5-mL centrifuge tube and placed in ice. RIPA buffer (70–100 μL) was added to the samples, and the tubes incubated for 30 min for cell lysis. Subsequently, the cells were further lysed using ultrasound. The supernatant was then centrifuged in a low-temperature centrifuge at 12000 RPM for 15 min. The supernatant was collected in a new centrifuge tube and the protein concentration was determined using the BCA method. Polyacrylamide gel was prepared according to the size of the target protein. After sodium dodecyl sulfate–polyacrylamide gel electrophoresis, the target protein was transferred to a PVDF membrane. The membrane was placed in 5% milk or bovine serum albumin for 1 h to block non-specific sites. After washing and the corresponding primary antibody was incubated at 4°C overnight. Then, the membrane was rinsed with 1×PBS-0.1% Tween three times, and mouse or rabbit secondary antibodies with corresponding primary antibody properties were incubated at room temperature for 1 h. Enhanced chemiluminescence solution was used to develop the protein bands, and the image was used to quantitatively analyze the optical density of the protein bands.

### Cell transfection

When the cell density was approximately 70%, a mixture containing the transfection reagent and plasmid was prepared according to the lipofectamine manufacturer’s instructions. After incubating for 15 min, the medium containing the plasmid–liposome complex was gently mixed with the cell supernatant. Transfected cells were collected for subsequent experiments 48 h after incubation at 37°C in a humidified 5% CO_2_ atmosphere.

### Human tissue and immunohistochemistry

Gingival tissue samples without gingival inflammation (healthy control group, n = 5) and gingival tissue samples with periodontal disease (deep pocket > 5mm) (Inflammation group, n = 5) were selected from routine oral care patients undergoing orthodontic treatment or periodontitis tooth extraction in the Stomatological Hospital of Xiamen Medical College. This study was approved by the Ethics Committee of the Stomatological Hospital of Xiamen Medical College (HS20200908001), and informed consent was obtained from each patient. Tissues were fixed with 4% formaldehyde at 4°C for 24 h, dehydrated, and embedded in paraffin wax. The embedded specimens were cut into 5-µm tissue slices and baked for 2 h in an oven at 65°C. Immunohistochemistry was performed as described previously (Xu et al., 2009). Primary antibodies against ITGB6 2746294 (Millipore, Billerica, MA, USA), pSTAT3 (Y705; Cell Signaling Technology, Danvers, MA,USA), and pFOXO1 (ab76055; Abcam, Cambridge, MA, USA) were used. The staining intensity in each section was measured using the cell counting function of the Image-Pro^®^ Plus software.

### Double luciferase reporter gene assay

A double luciferase reporter assay kit was used to detect the effect of FOXO1 and STAT3 plasmids on the ITGB6 gene promoter. The passive lysis buffer (PLB, 20 µL) was added to the cells plated in 96-well plate. After 15 min, 20 µL of PLB lysate was transferred into a 1.5-mL centrifuge tube. First, 20 µL of the LAR solution was added to the tube to determine the firefly luciferase value. The Renilla luciferase value was obtained by immediately adding 20 µL of STOP&Glo buffer. The firefly luciferase/Renilla luciferase intensity ratio was calculated for each group.

### Chromatin immunoprecipitation

The chromatin immunoprecipitation (ChIP) assay was performed using a ChIP kit (Cell Signaling Technology, Danvers, MA, USA). Cells were cultured to a density of 70%–80% (10-cm dish) collected after drug stimulation. Crosslinking was performed with 1% formaldehyde. After fixation, 10× glycine units were added to terminate the cross-linking process. After three times of washing with PBS, the sample was collected in a 1.5-mL centrifuge tube, and 1 µL of 100 mM PMSF and 1 µL of PIC were added. The sample was resuspended in 1 mL of ChIP-lysis buffer. Ultrasound treatment was performed 30 min after ice lysis to concentrate chromatin DNA fragments of 500–1000 bp. The sample was centrifuged at 12000 RPM at 4 °C for 10 min, and the supernatant was collected in a new centrifuge tube. After the DNA fragments were separated and purified, chromatin fragmentation was observed using 12% agarose gel electrophoresis. The number of immunoprecipitation (IP) and Ig G groups was calculated according to the concentration. Proteins were captured *via* immunoprecipitation using the corresponding primary antibody or control IgG and incubated overnight at 4°C. Further collection and purification of chromatin fragments was performed, followed by protein unlinking at 65°C. The precipitated DNA fragments were analyzed using PCR.

### Coimmunoprecipitation

Three groups of cells (IP-FOXO1 group, IP-STAT3 group, and IgG control group) were prepared using two 10-cm discs in each group. HaCaT cells were washed twice with pre-cooled PBS and then collected in a 1.5-mL centrifuge tube with a pre-cooled cell scraper. Subsequently, 1 mL of mild IP cracking solution was added, sloshed slowly for 15 min, and left to crack on ice for 1 h. After centrifugation at 14000 RPM for 15 min at 4°C, the supernatant was collected in a new centrifuge tube, 100 µL was removed from each set as input, and its concentration was determined using the BCA method. Each input group was supplemented with 25 µL of 5× loading buffer. This was boiled for 10 min and store at −20°C. After cleaning the protein A agarose twice with pre-cooled PBS, the protein A agarose with concentration of 50% was prepared. Protein A agarose (100 µL) was added to each centrifuge tube and incubated for 2 h in a suspension apparatus at 4°C. The IP group was incubated with the corresponding antibodies, and protein A agarose and incubated overnight under continuous rotation at 4°C. After centrifugation, the supernatant was carefully removed. Another 1 mL of IP lysate was added and washed three times for 3 min each, followed by centrifugation at 4°C at 3000 RPM for 3 min. After the last washing, 30 µL of 2× loading buffer was boiled and centrifuged to remove the precipitate, and the supernatant was collected as the protein sample of the IP group. Proteins released from the complex components were examined *via* SDS-PAGE and western blotting.

### Statistical analysis

The significance of differences between groups was determined using an unpaired two-tailed Student’s *t*-test and ANOVA using GraphPad Prism software (version 7.0). All experiments were independently repeated at least three times, and the differences were considered significant when *P < 0.05, **P < 0.01, and ***P < 0.001.

## Results

### Lipopeptide FSL-1 downregulates ITGB6 expression in epithelial cells

To elucidate the mechanism underlying ITGB6 downregulation in periodontitis, oral biofilm component FSL-1 was used as the stimulus.

**Figure 1:**
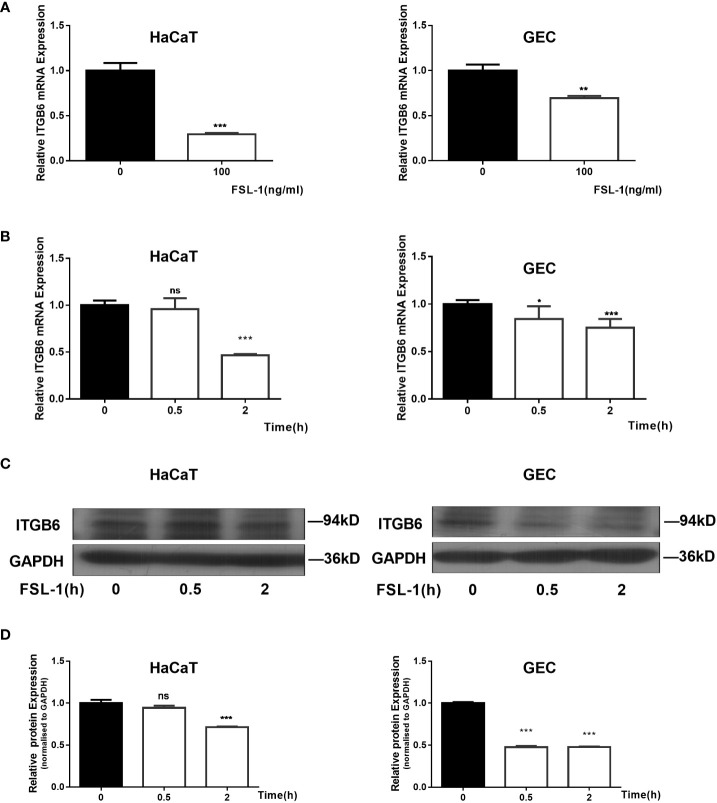
Suppression of ITGB6 by FSL-1 in human epithelial cells. **(A)** HaCaT (Left) or GECs (Right) were treated with 100 ng/mL FSL-1 for 2 h. *ITGB6* mRNA were then extracted, and the mRNA levels were measured by qRT-PCR. **(B)** HaCaT (Left) or GECs (Right) were treated with 100 ng/ml FSL-1 for indicated times, and the *ITGB6* mRNA expression levels were detected by qRT-PCR. **(C)** HaCaT or GECs were treated with 100 ng/mL FSL-1 for indicated times and the protein levels were assessed using western blotting. **(D)** Densitometric analysis was used to quantify protein bands. Data are presented as mean ± SEM from triplicates. no significance (ns), *P < 0.05, **P < 0.01, ***P < 0.001, comparison with time zero medium control cells.

As 100ng/mL FSL-1 has been reported to significantly inhibit ITGB6 expression in human GECs (Bi et al., 2017), we chose this concentration to investigate the mechanism of ITGB6 downregulation in epithelial cells. Epithelial HaCaT and GECs were exposed to FSL-1 (100 ng/mL) for the indicated times (0.5–2 h). ITGB6 mRNA and protein levels were detected using RT-qPCR and western blotting, respectively. The results showed that FSL-1 significantly downregulated ITGB6 mRNA expression in a time-dependent manner in both HaCaT and GECs (**Figures 1A, B**), with a 50% reduction in HaCaT and 30% reduction in GECs after 2 h of stimulation. FSL-1 also significantly attenuated ITGB6 protein production after 0.5–2 h of stimulation in HaCaT and GECs (**Figures 1C, D**). These results indicate that FSL-1 can reduce ITGB6 transcriptional expression in both the human epithelial cell line and primary derived GECs.

### FOXO1 and STAT3 binding sites are located in ITGB6 promoter

**Figure 2:**
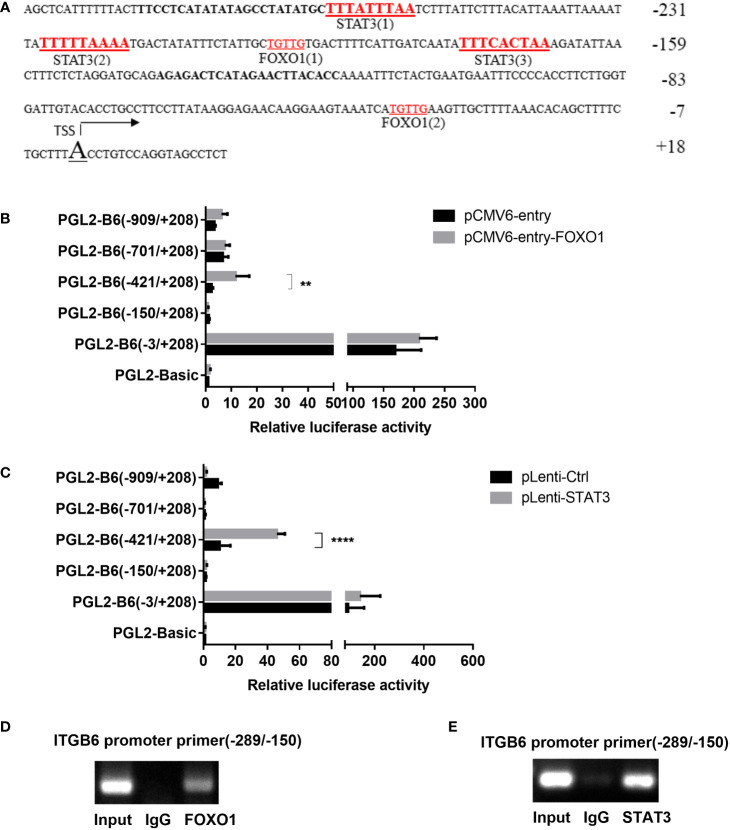
FOXO1 and STAT3 binding sites located at the ITGB6 promoter. **(A)** Potential binding sites at the ITGB6 promoter were predicted by the JASPER database. All numbers presented are relative to the TSS of the human *ITGB6* gene. There are three latent STAT3 binding sites and two FOXO1 binding sites between −26 and +18 of the *ITGB6* promoter. **(B, C)** Effect of FOXO1 **(B)** and STAT3 **(C)** overexpression on *ITGB6* promoter activity in HaCaT. Luciferase reporter constructs containing various lengths of the *ITGB6* promoter, or the empty plasmid pGL2-basic, were co-transfected with pRL-TK and with either FOXO1 or STAT3-overexpressing plasmid into HaCaT cells. Luciferase activity was measured at 48 h after transfection. The luciferase value of the pGL2-basic was set to a value of one. Relative promoter activities are presented on the right (mean ± SEM, at least three independent experiments) and were analyzed using one-way ANOVA. **(D, E)** Measurement of FOXO1 and STAT3 binding to the ITGB6 promoter. ChIP assay was performed using anti-FOXO1 antibody **(D)**, anti-STAT3 antibody **(E)**, or IgG as a control in HaCaT cells. Input and immunoprecipitated DNA was then amplified by PCR using primer pairs covering FOXO1 and STAT3 binding sites from −289 to −150 bp. The associated *ITGB6* promoter DNA was amplified using PCR and resolved using agarose electrophoresis. **P < 0.01 and ****P < 0.0001.

As ITGB6 expression is transcriptionally regulated and the transcription factors FOXO1 and STAT3 are involved in the inflammatory response, we hypothesized that FOXO1 and STAT3 are involved in ITGB6 transcription in epithelial cells. Sequence analysis predicted several putative transcription factor FOXO1 and STAT3 binding sites in the −289 to −150 region of the ITGB6 promoter (**Figure 2A**). Overexpression of FOXO1 or STAT3 significantly upregulated the activity of the pGL2-B6(−421/+208) construct but had no effect on the activity of the pGL2-B6(−150/+208) construct, suggesting that the region between −421 and −150 bp upstream of the translational start codon may contain binding sites for the transcription factors FOXO1 and STAT3 (**Figures 2B, C**). To determine whether cellular FOXO1 and STAT3 can bind to the ITGB6 promoter, a ChIP assay was performed and assayed using PCR targeting the −421 to −150 region of the ITGB6 promoter. ChIP with an antibody against FOXO1 and STAT3 resulted in marked enrichment of the ITGB6 promoter DNA compared to the control IgG (**Figures 2D, E**). These results suggest that FOXO1 and STAT3 could bind to the region located at positions −421 to −150 of the human ITGB6 promoter *in vivo* and may be involved in human ITGB6 promoter transcriptional activity.

### FOXO1 is involved in FSL-1-mediated ITGB6 downregulation

**Figure 3:**
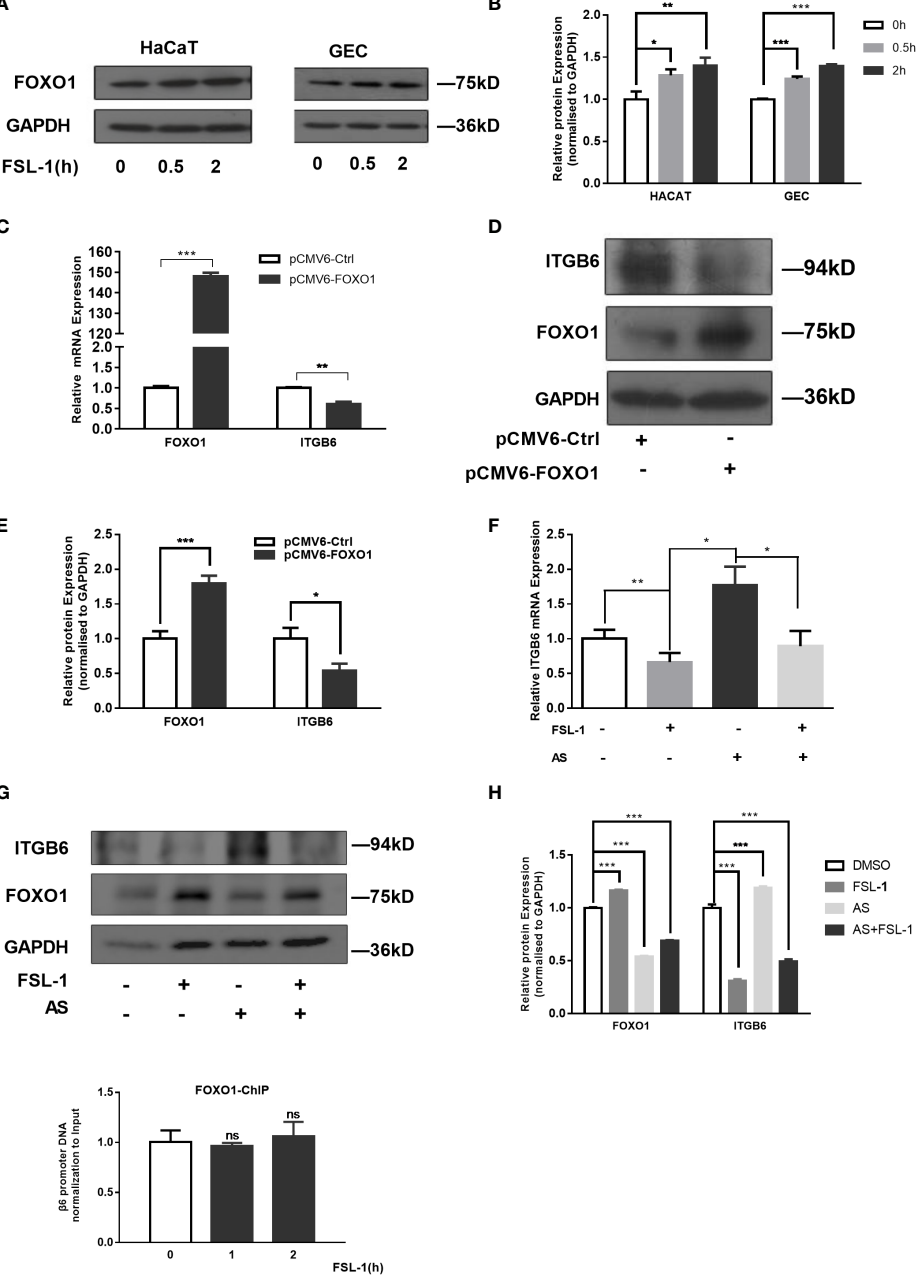
FOXO1 is involved in ITGB6 suppression by FSL-1 in HaCaT and GECs. Serum-starved HaCaT and GECs were treated with FSL-1 (100 ng/mL) for 0.5–2 h, and the total FoxO1 **(A)** protein expression levels were analyzed using western blotting. Protein loading was verified using GAPDH. **(B)** Densitometric analysis was used to quantify protein bands. Data are presented as mean ± SEM from triplicates, comparised with time zero medium control cells **(C)** HaCaT cells were transfected with pCMV6-Ctrl or pCMV6-FOXO1 for 48 h. Total RNA was extracted, and the mRNA expression of *ITGB6* was detected by RT-qPCR. Data are shown as the mean ± SEM of triplicates and comparisoned with the pCMV6-Ctrl group. **(D)** Protein expression levels were analyzed using Western blotting. Protein loading was verified using GAPDH. **(E)** Densitometric analysis was used to quantify protein bands. Data are presented as mean ± SEM from triplicates and compared with pCMV6-Ctrl group. The effect of the FSL-1 inhibitor AS1842856 (AS) on the expression level of *ITGB6* mRNA **(F)** and protein **(G)**. HaCaT cells were exposed to FSL-1 (100 ng/mL) for 2 h with or without pre-incubation with AS (1 μM) for 0.5 h. Final concentrations of DMSO were kept constant for all treatment conditions. *ITGB6* mRNA levels were determined using qRT-PCR. Data are shown as mean ± SEM of triplicates. Protein levels of ITGB6, FOXO1, and GAPDH (loading control) were measured using western blotting. The blots are representative of three independent experiments with similar results **(G)**. **(H)** Densitometric analysis was used to quantify protein bands. Data are presented as mean ± SEM from triplicates. **(I)** Measurement of FOXO1 binding to ITGB6 promoter. HaCaT cells were treated with FSL-1 at the indicated times, and the binding of FOXO1 to the *ITGB6* promoter was detected by ChIP assay. The associated *ITGB6* promoter DNA was measured using qPCR. The results were normalized to the input control. Data are shown as mean ± SEM of triplicates. no significance (ns), compared to the time zero. *P < 0.05, **P < 0.01, ***P < 0.001.

To explore whether FOXO1 is involved in FSL-1-decreased ITGB6 expression in HaCaT and GECs, we first examined the effect of FSL-1 on FOXO1 expression using western blotting. As shown in **Figure 3**, FSL-1 significantly upregulated FOXO1 (**Figures 3A, B**) protein levels in a time-dependent manner in both HaCaT and GECs. Furthermore, overexpression of FOXO1 significantly decreased the expression level of mRNA (about 40%, P < 0.01) and protein of ITGB6 (**Figures 3C–E**). In addition, the FSL-1 inhibitor AS1842856 significantly reversed the expression of mRNA (about 20%, P < 0.05) and ITGB6 protein (**Figures 3F–H**), which was inhibited by FSL-1. These results indicated that FOXO1 is involved in the downregulation of ITGB6 expression.

Next, a ChIP assay was performed to further clarify whether FOXO1 mediates the effect of FSL-1 on ITGB6 transcription *via* increased binding to the ITGB6 promoter *in vivo.* HaCaT cells were treated with FSL-1 (100 ng/mL) for 1–2 h, and whole-cell chromatin was extracted. As shown in **Figure 3I**, no increase in the binding of FOXO1 to the ITGB6 promoter was detected after FSL-1 stimulation, suggesting that FOXO1 involvement in ITGB6 downregulation was not mediated by increased binding to the ITGB6 promoter site.

### STAT3 suppression is involved in ITGB6 downregulation by FSL-1

**Figure 4:**
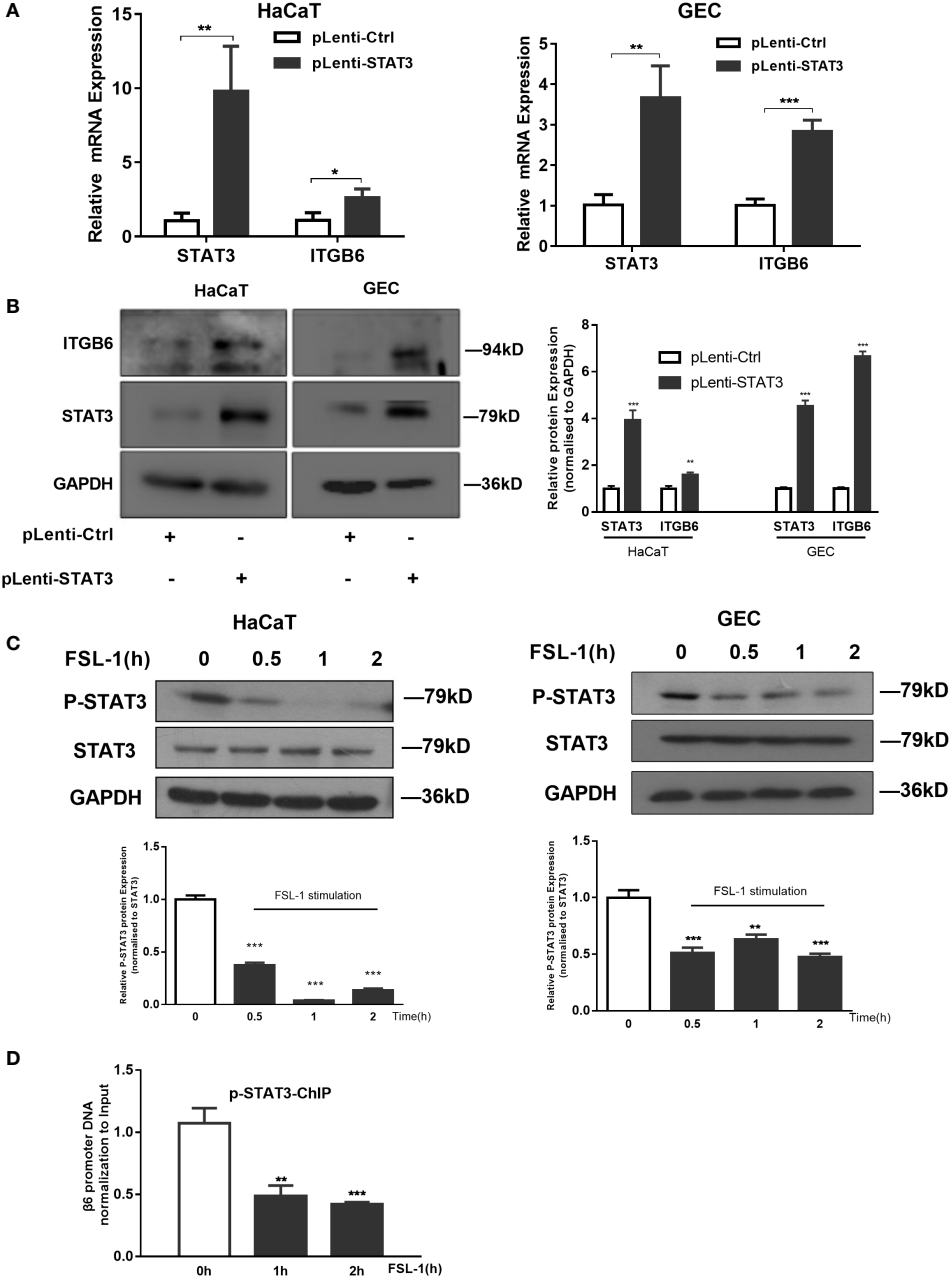
STAT3 suppression is involved in FSL-1 mediated ITGB6 downregulation. **(A, B)** STAT3 is involved in ITGB6 transcriptional regulation in HaCaT and GECs. Serum-starved HaCaT and GECs were transfected with the control vectors pLenti-Ctrl or pLenti-STAT3 for 48 h. The mRNA expression of STAT3 and ITGB6 was measured by qRT-PCR **(A)** Data are presented as mean ± SEM from triplicates and compared with pLenti-Ctrl. The protein expression of ITGB6 and STAT3 in HaCaT and GECs was measured using western blotting (left). Densitometric analysis was used to quantify protein bands (right). Data are presented as mean ± SEM from triplicates and compared with pLenti-Ctrl group. **(C)** FSL-1 inhibits STAT3 activation in HaCaT and GECs. Serum-starved HaCaT and GECs were treated with FSL-1 (100 ng/mL) for the indicated times (0.5–2 h). Whole-cell extracts were prepared for western blot analysis of phosphorylated STAT3, STAT3, and GAPDH (loading control). Densitometric analysis was used to quantify protein bands (lower panel). Data are presented as mean ± SEM from triplicates and compared with time zero group. **(D)** FSL-1 inhibited phosphorylated STAT3 binding to the *ITGB6* promoter. HaCaT cells were treated with FSL-1 (100 ng/mL) for the indicated times (1–2 h), and the binding of STAT3 to the *ITGB6* promoter was detected using ChIP assay. The associated *ITGB6* promoter DNA was amplified using qPCR. Data are shown as the mean ± SEM of triplicate experiments and comparison with time zero medium control cells. *P < 0.05, **P < 0.01, ***P < 0.001.

To explore the role of STAT3 in FSL-1-decreased ITGB6 transcription, we first examined the effect of STAT3 on ITGB6 expression in HaCaT and GECs. As shown in **Figure 4**, STAT3 overexpression increased the expression levels of ITGB6 mRNA and protein in both HaCaT and GECs (**Figures 4A, B**). These results indicated that STAT3 may be involved in the upregulation of ITGB6 expression.

Next, we examined the effect of FSL-1 on STAT3 activation using western blotting. As shown in **Figure 4**, FSL-1 significantly decreased STAT3 phosphorylation in both cell lines from 0.5 h onwards (**Figure 4C**). Furthermore, the ChIP assay results showed that FSL-1 stimulation inhibited the binding of phosphorylated STAT3 to the ITGB6 promoter site from 1 h onwards (**Figure 4D**). Overall, these results suggest that inhibition of phosphorylated STAT3 binding to the ITGB6 promoter is involved in the FSL-1-mediated ITGB6 transcriptional downregulation.

### Transcription factor FOXO1 interacts with STAT3 to mediate the effect of FSL-1 on ITGB6 downregulation

**Figure 5:**
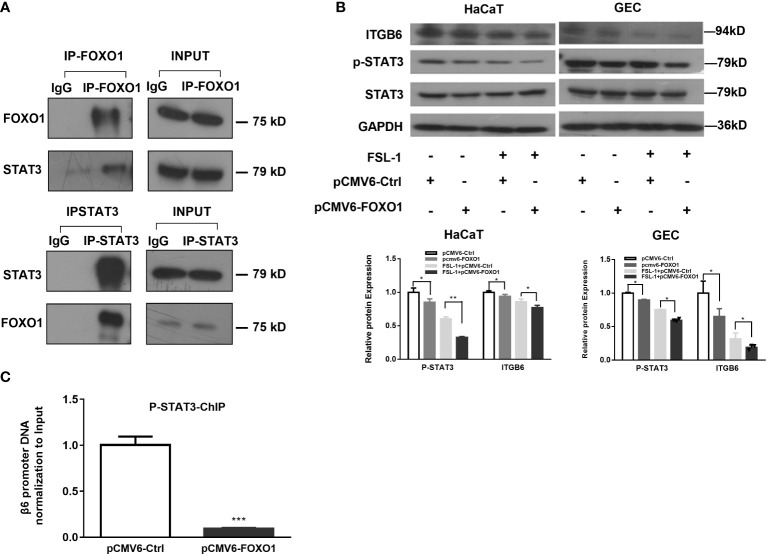
FOXO1-inhibited STAT3 activation is involved in FSL-1-mediated ITGB6 downregulation. **(A)** The interaction of STAT3 and FOXO1 was detected *via* co-immunoprecipitation in HaCaT cells. Cell lysate was incubated with FOXO1, STAT3 antibodies or control IgG. The corresponding interacting protein was then detected by western blotting using antibody against STAT3, FOXO1, or IgG. Cell lysate (5%) was used as input positive control. **(B)** Overexpression of FOXO1 inhibited STAT3 activation and ITGB6 expression. HaCaT cells were transfected with pCMV6-Ctrl or pCMV6-FOXO1 plasmids for 24 h before treatment with or without FSL-1 (100 ng/mL) for 2 h. The protein expression of FOXO1, STAT3, p-STAT3, and ITGB6 was analyzed using western blotting. **(C)** FOXO1 overexpression inhibited phosphorated STAT3 binding to the ITGB6 promoter. HaCaT cells were transfected with pCMV6-Ctrl or pCMV6-FOXO1 plasmids for 24 h. The binding of phosphorated STAT3 to the ITGB6 promoter was detected by ChIP assay. The associated ITGB6 promoter DNA was amplified by qPCR using primers covering the potential STAT3-binding sites from −289/−150 of ITGB6 promoter. The input was 5% of the total cell lysate. Data are shown as mean ± SEM of triplicates and compared, V6-Ctrl group. *P < 0.05, **P < 0.01, ***P < 0.001.

As FOXO1 negatively regulates leptin-induced pro-opiomelanocortin (POMC) transcription through its direct interaction with STAT3 (Ma et al., 2015), we speculated that FOXO1 may mediate the negative effect of FSL-1 on ITGB6 expression *via* interaction with STAT3. To test this possibility, we first performed co-immunoprecipitation experiments to assess the interaction between FOXO1 and STAT3, and the results confirmed the interaction between FOXO1 and STAT3 (**Figure 5A**).

Moreover, FOXO1 overexpression attenuated STAT3 phosphorylation. In addition, incubation with FSL-1 after FOXO1 overexpression further downregulated STAT3 phosphorylation levels, suggesting a synergistic effect on STAT3 phosphorylation level by FSL-1 incubation and FOXO1 overexpression (**Figure 5B**). Next, we performed a ChIP assay to determine the effect of FOXO1 overexpression on the binding of phosphorylated STAT3 (p-STAT3) to the ITGB6 promoter. The results (**Figure 5C**) showed that FOXO1 overexpression significantly inhibited the binding of p-STAT3 to the ITGB6 promoter.

Thus far, the *in vitro* data strongly suggest that transcription factor FOXO1 is involved in regulating the effect of FSL-1 on ITGB6 downregulation by inhibiting STAT3 activation and reducing the binding of p-STAT3 to the ITGB6 promoter region.

**Figure 6:**
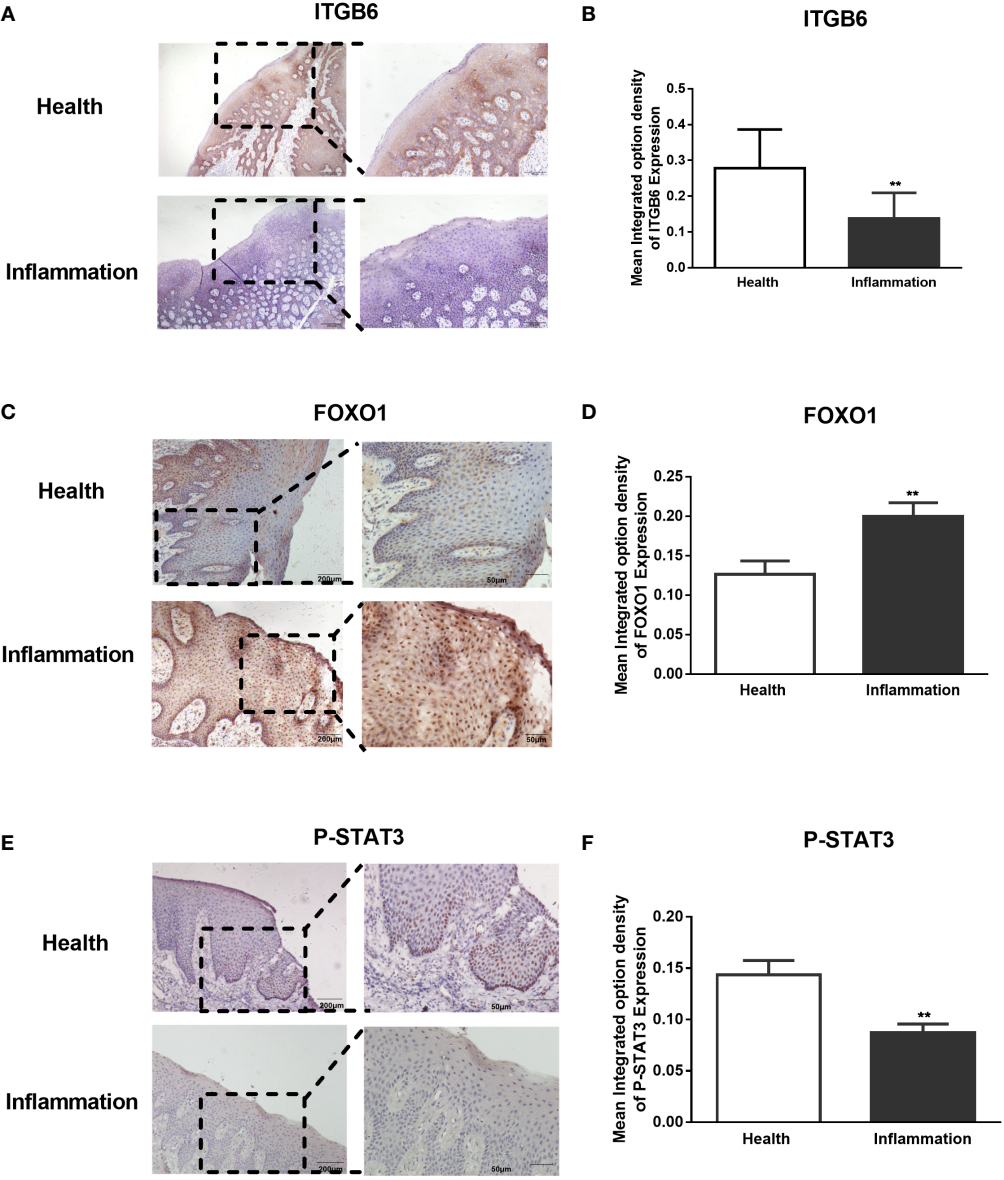
Expression of ITGB6, FoxO1, and phosphorated STAT3 in human gingival epithelium. Paraffin-embedded human gingival epithelia were obtained from patients who had undergone tooth extraction for periodontitis treatment (n = 5) or orthodontic treatment as healthy controls (n=5). Immunohistochemistry for ITGB6, phosphorated STAT3, and FOXO1 was performed. Representative sections of healthy gingival epithelium showing strong staining of ITGB6 in the cytosol **(A)** and phosphorylated STAT3 in the nucleus **(E)**, whereas both molecules were barely expressed in periodontal disease specimens. In contrast, a representative section of inflammatory gingival epithelium showed strong staining of FOXO1 in both the nucleus and cytoplasm, whereas minor staining was detected in healthy gingival epithelium **(C)**. **(A)** Low power scale bar: 200 μm. High power scale bar: 100 μm. **(C–E)** Low power scale bar: 200 μm. High power scale bar: 50 μm. **(B, D, F)** Quantitative statistics of immunostaining were analyzed using Image Pro-Plus 6.0. The average integrated optical density (IOD) of both healthy and inflamed tissues was analyzed. Five regions of the epithelial tissue were randomly selected for the average value. Data are shown as the mean ± SEM of triplicate experiments and compared to healthy gingival epithelium. **P < 0.01.

To confirm the role of FOXO1 and STAT3 and its relation to ITGB6 expression *in vivo*, we performed immunohistochemistry to detect the expression of ITGB6, FOXO1, and STAT3 using sequencing slices from healthy human gingival tissue and gingival epithelium of patients with periodontitis. First, we confirmed the reduction of ITGB6 expression level in periodontitis samples as compared with that in the healthy controls (**Figures 6A, B**). As shown in **Figures 6E, F**, p-STAT3 was expressed in the nucleus of healthy gingival epithelium but was minimally detected in inflammatory tissue. On the contrary, FOXO1 was strongly detected in the inflammatory epithelium, both in the nucleus and cytoplasm, and was weakly detected in healthy gingival epithelium (**Figures 6C, D**). These results suggest that FOXO1 interacts with STAT3 and may be involved in the downregulation of ITGB6 in the inflammatory epithelium associated with periodontitis.

## Discussion

The presence of ITGB6 is essential for periodontal health, as its low expression in the gingival epithelium is related to the development of periodontal diseases (Ghannad et al., 2008). Although previous research has confirmed that periodontal bacteria or pathogens may cause ITGB6 repression in periodontitis (Bi et al., 2017), limited work has been performed to further investigate the transcriptional mechanism of ITGB6 downregulation in periodontitis. In the present study, we found that the interaction of the transcription factors FOXO1 and STAT3 mediated periodontitis-related lipopeptide FSL-1-induced ITGB6 downregulation in human epithelial cells. While EGFR–ERK signaling pathways have previously been implicated in ITGB6 suppression in periodontal inflammation (Bi et al., 2019), the critical involvement of the transcription factors FOXO1 and STAT3 in ITGB6 suppression by oral bacterial biofilm components is a novel finding.

It is generally accepted that gene expression is critically governed by various transcription factors in a cell- and stimulus-specific manner. However, the composition of periodontal bacteria or pathogens that cause periodontitis is complex, and it is difficult to determine the effect of these pathogens on ITGB6 expression. Therefore, we selected a single microbial biofilm component, FSL-1, to study ITGB6 transcriptional regulation. FSL-1 is the N-terminal lipopeptide of the cell membrane lipoprotein LP44 produced by the common oral bacterium *M. salivarium*, which preferentially inhabits the gingival sulci and is suspected to play an etiological role in periodontal diseases (Shibata et al., 2000). Our results showed that FSL-1 could suppress ITGB6 expression in both commercial epithelial cell lines and primary GECs, which is consistent with the results of a previous study (Bi et al., 2017). Moreover, the effect of FSL-1 on ITGB6 suppression is similar to that of oral bacterial biofilm extracts (Bi et al., 2017). FSL-1 was then used as a stimulator to investigate the transcriptional regulation mechanism of ITGB6 suppression associated with periodontitis in the current study.

Accumulating evidence indicates that FOXO1 activity can be induced by periodontal pathogens and plays a crucial role in periodontal homeostasis and disease (Ren et al., 2021). Wang et al. showed that FOXO1 accumulates in the nucleus of *Porphyromonas gingivalis*-infected cells (Wang et al., 2015) and is involved in gene expression related to cell death, barrier function, differentiation, and inflammation (Li et al., 2013). In our study, we verified that FSL-1 significantly upregulated the expression of FOXO1. In addition, upregulation of FOXO1 downregulated ITGB6 expression, whereas blocking FOXO1 expression inhibited the downregulation of ITGB6 by FSL-1. These results indicated the involvement of FOXO1 in FSL-1-induced ITGB6 downregulation. Transcription factors normally regulate gene expression by binding to the target gene promoter. However, FOXO1 binding to the ITGB6 promoter did not increase upon FSL-1 stimulation. This suggested that FSL-1-induced FOXO1 expression may indirectly regulate ITGB6 transcription *via* a different mechanism such as protein–protein interaction. Previous studies have confirmed that FOXO1 may regulate the expression of target genes without directly binding to DNA. A FOXO1 mutant that lacks DNA-binding activity can regulate target gene expression (Ramaswamy et al., 2002). In addition, FOXO1 can regulate target gene transcription through direct interaction with other transcription factors, such as STAT3 (Yang et al., 2009; Oh et al., 2012; Ma et al., 2015; Zhang et al., 2021). STAT3 is a critical transcription factor in inflammatory responses and induces the expression of a large array of inflammatory mediators (Hillmer et al., 2016). We previously reported that the promoter region of the human ITGB6 gene contains STAT3 binding sites, and the basal rate of ITGB6 expression in oral squamous cell carcinoma cells is primarily defined by STAT3 (Xu et al., 2015). Therefore, we speculated that the reciprocal action of STAT3 and FOXO1 might regulate FSL-1-induced ITGB6 downregulation.

Being a pivotal inflammatory transcription factor, STAT3 activation has been shown to mediate the biological effects of several oral proinflammatory factors such as *P. gingivalis* (Ma et al., 2021) and LPS (Zhang et al., 2021). However, it is still unclear whether STAT3 plays a role in FSL-1-induced cellular effects in periodontitis. Our results demonstrated for the first time that FSL-1 stimulation reduced STAT3 phosphorylation levels in both HaCaT and GECs. FSL-1 stimulation also inhibits the binding of phospho-STAT3 to the ITGB6 promoter. These results indicate that FSL-1 downregulates ITGB6 expression in GECs by inhibiting STAT3 activation and binding to the ITGB6 promoter.

**Figure 7:**
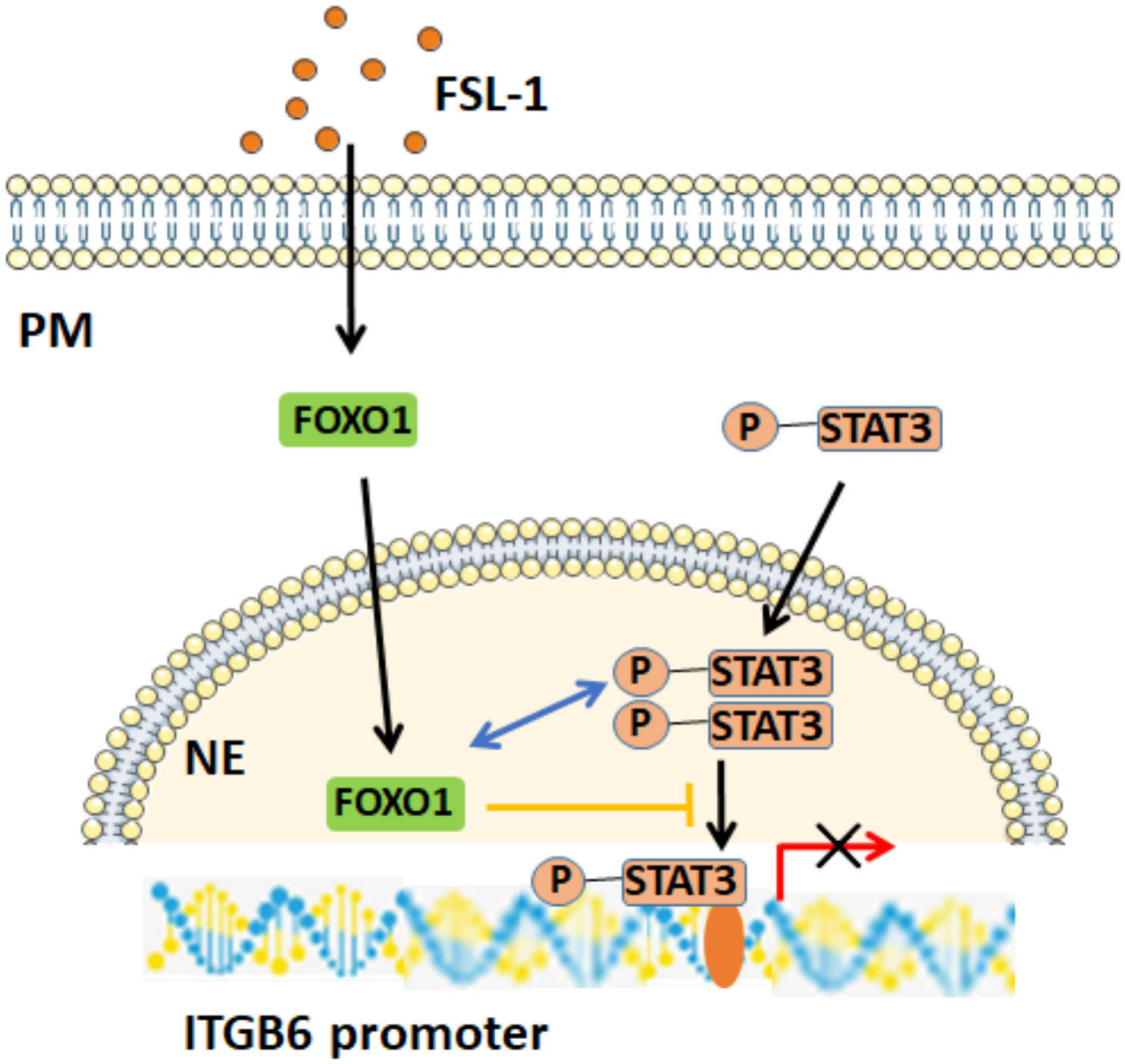
Potential mechanism of FOXO1-mediated FSL-1-induced ITGB6 downregulation. FSL-1 increases FOXO1 expression, and FOXO1 interacts with p-STAT3 in the nucleus, prevents the binding of p-STAT3 to the ITGB6 promoter, and consequently inhibits STAT3-mediated ITGB6 transcriptional expression. *PM*, plasma membrane; *NE*, nuclear envelope.

We also determined how FOXO1 interfered with STAT3 to regulate ITGB6 suppression by FSL-1. First, our co-IP experiments confirmed the direct interaction of FOXO1 with STAT3. Next, overexpression of FOXO1 further attenuated STAT3 activation and its binding to the ITGB6 promoter. These results are consistent with the notion that FOXO1 inhibits STAT3 activity by preventing STAT3 from interacting with the ITGB6 promoter DNA. Furthermore, the reciprocal action of STAT3 and FOXO1 on ITGB6 downregulation was also confirmed by the immunostaining of the inflammatory epithelium associated with periodontitis. Together, these data support our proposed model of a potential mechanism of how FSL-1-induced FOXO1 inhibits ITGB6 transcription regulation; with increasing amount of FOXO1 expression, FOXO1 binds to phospho-STAT3 in the nucleus, prevents STAT3 from interacting with the ITGB6 promoter, and consequently, inhibits ITGB6 transcription (**Figure 7**).

In conclusion, the current study demonstrates that FOXO1 is involved in FSL-1-mediated ITGB6 downregulation in HaCaT and GECs. The FOXO1 inhibitory effect on ITGB6 expression was a consequence of its interaction with STAT3 rather than binding to the ITGB6 promoter site. FOXO1 attenuated STAT3 activation and binding to the ITGB6 promoter, which resulted in FSL-1-induced downregulation of ITGB6. Therefore, the interaction of FOXO1 and STAT3 may contribute to periodontitis progression by inhibiting ITGB6 expression. Targeting the FOXO1–STAT3 signaling axis may be an effective method for treating periodontal diseases.

## Data availability statement

The raw data supporting the conclusions of this article will be made available by the authors, without undue reservation.

## Ethics statement

The studies involving human participants were reviewed and approved by the Ethics Committee of the Stomatological Hospital of Xiamen Medical College (HS20200908001). The patients/participants provided their written informed consent to participate in this study.

## Author contributions

MX contributed to conception, analysis, and interpretation, drafted and critically revised the manuscript; JH and FZ, contributed to data acquisition, analysis, and interpretation the manuscript; KS and FL contributed to conception and critically revised the manuscript; XD contributed to conception and design, and drafted the manuscript. All authors gave final approval and agreed to be accountable for all aspects of the work.

## Funding

This work was supported by National Natural Science Foundation of China (NO. 82071128, 82270988, 81771079), the Fujian Provincial Health Technology Project (2020-CXB055) and the Xiamen Health Commission project (2022D006).

## Acknowledgments

We would like to thank Editage (www.editage.cn) for English language editing.

## Conflict of interest

The authors declare that the research was conducted in the absence of any commercial or financial relationships that could be construed as a potential conflict of interest.

## Publisher’s note

All claims expressed in this article are solely those of the authors and do not necessarily represent those of their affiliated organizations, or those of the publisher, the editors and the reviewers. Any product that may be evaluated in this article, or claim that may be made by its manufacturer, is not guaranteed or endorsed by the publisher.

## References

[B1] Ahmad R. Shihab P. K. Jasem S. Behbehani K. (2014). FSL-1 induces MMP-9 production through TLR-2 and NF-kappaB/AP-1 signaling pathways in monocytic THP-1 cells. Cell Physiol. Biochem. 34, 929–942. doi: 10.1159/000366310 25200491

[B2] Ansar M. Jan A. Santos-Cortez R. L. Wang X. Suliman M. Acharya A.. (2016). Expansion of the spectrum of ITGB6-related disorders to adolescent alopecia, dentogingival abnormalities and intellectual disability. Eur. J. Hum. genetics: EJHG 24, 1223–1227. doi: 10.1038/ejhg.2015.260 26695873 PMC4970676

[B3] Bi J. Dai J. Koivisto L. Larjava M. Bi L. Hakkinen L.. (2019). Inflammasome and cytokine expression profiling in experimental periodontitis in the integrin beta6 null mouse. Cytokine 114, 135–142. doi: 10.1016/j.cyto.2018.11.011 30467097

[B4] Bi J. Koivisto L. Dai J. Zhuang D. Jiang G. Larjava M.. (2019). Epidermal growth factor receptor signaling suppresses alphavbeta6 integrin and promotes periodontal inflammation and bone loss. J. Cell Sci. 133 (5), jcs236588. doi: 10.1242/jcs.236588. 31722981

[B5] Bi J. Koivisto L. Pang A. Li M. Jiang G. Aurora S.. (2017). Suppression of alphavbeta6 integrin expression by polymicrobial oral biofilms in gingival epithelial cells. Sci. Rep. 7, 4411. doi: 10.1038/s41598-017-03619-7 28667248 PMC5493688

[B6] Breuss J. M. Gillett N. Lu L. Sheppard D. Pytela R. (1993). Restricted distribution of integrin beta 6 mRNA in primate epithelial tissues. J. Histochem Cytochem 41, 1521–1527. doi: 10.1177/41.10.8245410 8245410

[B7] Ghannad F. Nica D. Fulle M. I. Grenier D. Putnins E. E. Johnston S.. (2008). Absence of alphavbeta6 integrin is linked to initiation and progression of periodontal disease. Am. J. Pathol. 172, 1271–1286. doi: 10.2353/ajpath.2008.071068 18385522 PMC2329836

[B8] Gomes-Filho I. S. Cruz S. S. D. Trindade S. C. Passos-Soares J. S. Carvalho-Filho P. C. Figueiredo A.. (2020). Periodontitis and respiratory diseases: A systematic review with meta-analysis. Oral. Dis. 26, 439–446. doi: 10.1111/odi.13228 31715080

[B9] Hickey N. A. Shalamanova L. Whitehead K. A. Dempsey-Hibbert N. van der Gast C. Taylor R. L. (2020). Exploring the putative interactions between chronic kidney disease and chronic periodontitis. Crit. Rev. Microbiol. 46, 61–77. doi: 10.1080/1040841X.2020.1724872 32046541

[B10] Hillmer E. J. Zhang H. Li H. S. Watowich S. S. (2016). STAT3 signaling in immunity. Cytokine Growth Factor Rev. 31, 1–15. doi: 10.1016/j.cytogfr.2016.05.001 27185365 PMC5050093

[B11] Kedjarune U. Pongprerachok S. Arpornmaeklong P. Ungkusonmongkhon K. (2001). Culturing primary human gingival epithelial cells: comparison of two isolation techniques. J. Craniomaxillofac Surg. 29, 224–231. doi: 10.1054/jcms.2001.0229 11562092

[B12] Kocher T. Konig J. Borgnakke W. S. Pink C. Meisel P. (2018). Periodontal complications of hyperglycemia/diabetes mellitus: Epidemiologic complexity and clinical challenge. Periodontol 2000 78, 59–97. doi: 10.1111/prd.12235 30198134

[B13] Li S. Dong G. Moschidis A. Ortiz J. Benakanakere M. R. Kinane D. F.. (2013). P. gingivalis modulates keratinocytes through FOXO transcription factors. PloS One 8, e78541. doi: 10.1371/journal.pone.0078541 24265696 PMC3827038

[B14] Ma W. Fuentes G. Shi X. Verma C. Radda G. K. Han W. (2015). FoxO1 negatively regulates leptin-induced POMC transcription through its direct interaction with STAT3. Biochem. J. 466, 291–298. doi: 10.1042/BJ20141109 25510553

[B15] Maiese K. Chong Z. Z. Hou J. Shang Y. C. (2009). The “O” class: crafting clinical care with FoxO transcription factors. Adv. Exp. Med. Biol. 665, 242–260. doi: 10.1007/978-1-4419-1599-3_18 20429429 PMC2863142

[B16] Ma L. Liu H. Wang X. Jiang C. Yao S. Guo Y.. (2021). CXXC5 orchestrates Stat3/Erk/Akt signaling networks to modulate p. gingivalis-elicited autophagy cementoblasts Biochim. Biophys. Acta Mol. Cell Res. 1868, 118923. doi: 10.1016/j.bbamcr.2020.118923 33285176

[B17] Meecham A. Marshall J. F. (2020). The ITGB6 gene: its role in experimental and clinical biology. Gene: X 5, 100023. doi: 10.1016/j.gene.2019.100023 34493369

[B18] Oh H. M. Yu C. R. Dambuza I. Marrero B. Egwuagu C. E. (2012). STAT3 protein interacts with class O forkhead transcription factors in the cytoplasm and regulates nuclear/cytoplasmic localization of FoxO1 and FoxO3a proteins in CD4(+) T cells. J. Biol. Chem. 287, 30436–30443. doi: 10.1074/jbc.M112.359661 22761423 PMC3436293

[B19] Ramaswamy S. Nakamura N. Sansal I. Bergeron L. Sellers W. R. (2002). A novel mechanism of gene regulation and tumor suppression by the transcription factor FKHR. Cancer Cell 2, 81–91. doi: 10.1016/S1535-6108(02)00086-7 12150827

[B20] Ren L. Yang J. Wang J. Zhou X. Liu C. (2021). The roles of FOXO1 in periodontal homeostasis and disease. J. Immunol. Res. 2021, 5557095. doi: 10.1155/2021/5557095 33860060 PMC8026307

[B21] Sanz M. Marco Del Castillo A. Jepsen S. Gonzalez-Juanatey J. R. D’Aiuto F. Bouchard P.. (2020). Periodontitis and cardiovascular diseases: Consensus report. J. Clin. Periodontol 47, 268–288. doi: 10.1111/jcpe.13189 32011025 PMC7027895

[B22] Shibata K. Hasebe A. Into T. Yamada M. Watanabe T. (2000). The n-terminal lipopeptide of a 44-kDa membrane-bound lipoprotein of mycoplasma salivarium is responsible for the expression of intercellular adhesion molecule-1 on the cell surface of normal human gingival fibroblasts. J. Immunol. 165, 6538–6544. doi: 10.4049/jimmunol.165.11.6538 11086096

[B23] Teles F. Collman R. G. Mominkhan D. Wang Y. (2022). Viruses, periodontitis, and comorbidities. Periodontol 2000 89, 190–206. doi: 10.1111/prd.12435 35244970

[B24] van der Vos K. E. Coffer P. J. (2008). FOXO-binding partners: it takes two to tango. Oncogene 27, 2289–2299. doi: 10.1038/onc.2008.22 18391971

[B25] Wang Q. Sztukowska M. Ojo A. Scott D. A. Wang H. Lamont R. J. (2015). FOXO responses to porphyromonas gingivalis in epithelial cells. Cell. Microbiol. 17, 1605–1617. doi: 10.1111/cmi.12459 25958948 PMC4624012

[B26] Xu M. Chen X. Yin H. Yin L. Liu F. Fu Y.. (2015). Cloning and characterization of the human integrin beta6 gene promoter. PloS One 10, e0121439. doi: 10.1371/journal.pone.0121439 25816241 PMC4376883

[B27] Xu M. Y. Porte J. Knox A. J. Weinreb P. H. Maher T. M. Violette S. M.. (2009). Lysophosphatidic acid induces {alpha}v{beta}6 integrin-mediated TGF-{beta} activation *via* the LPA2 receptor and the small G protein g{alpha}q. Am. J. Pathol. 174, 1264–1279. doi: 10.2353/ajpath.2009.080160 19147812 PMC2671359

[B28] Yang G. Lim C. Y. Li C. Xiao X. Radda G. K. Li C.. (2009). FoxO1 inhibits leptin regulation of pro-opiomelanocortin promoter activity by blocking STAT3 interaction with specificity protein 1. J. Biol. Chem. 284, 3719–3727. doi: 10.1074/jbc.M804965200 19049975

[B29] Zemedikun D. T. Chandan J. S. Raindi D. Rajgor A. D. Gokhale K. M. Thomas T.. (2021). Burden of chronic diseases associated with periodontal diseases: a retrospective cohort study using UK primary care data. BMJ Open 11, e048296. doi: 10.1136/bmjopen-2020-048296 PMC8689170 34924359

[B30] Zhang W. Bai S. S. Zhang Q. Shi R. L. Wang H. C. Liu Y. C.. (2021). Physalin b reduces abeta secretion through down-regulation of BACE1 expression by activating FoxO1 and inhibiting STAT3 phosphorylation. Chin. J. Nat. Med. 19, 732–740. doi: 10.1016/S1875-5364(21)60090-0 34688463

[B31] Zhang X. Zhang X. Qiu C. Shen H. Zhang H. He Z.. (2021). The imbalance of Th17/Treg *via* STAT3 activation modulates cognitive impairment in p. gingivalis LPS-induced periodontitis mice. J. Leukoc. Biol. 110, 511–524. doi: 10.1002/JLB.3MA0521-742RRR 34342041

